# Mechanisms of Latino Migrant Retention: Lived Experiences From the Third Age and Implications for Health

**DOI:** 10.1093/geroni/igaf122.1387

**Published:** 2025-12-31

**Authors:** Sarah Salas, Michal Engelman, Johanna Nunez

**Affiliations:** University of Wisconsin–Madison, Madison, Wisconsin, United States; University of Wisconsin-Madison, Madison, Wisconsin, United States; University of Wisconsin–Madison, Madison, Wisconsin, United States

## Abstract

Recent decades have seen significant growth in immigrant populations in non-traditional U.S destinations. Latinos are Wisconsin’s largest minority group at 8% of the population; immigrants comprise 27% of this group. As most research on immigrant acculturation focuses on those in early and midlife, the experiences of older Latino immigrants remain unclear. We conducted semi-structured life history interviews with 33 Latino immigrants aged 60 years or older living in Wisconsin. Our analysis examines how immigrants’ varied backgrounds shape their migration experiences, and how they relate to their health outcomes in later life. We draw on theories of immigrant identity formation and cultural ties across countries to examine Latinos’ lived experiences and migration histories. We find that reasons for migration (including loose social ties, family reunification, and health concerns) interplay with immigrants’ attitudes towards living in the U.S. to influence one’s retention pathway. Transnational older adults have mixed dispositions about being in the U.S. and can be on either constrained or classic retention pathways. While constrained retention involves external motivators for staying in the U.S. and largely includes those who wish to return to their home country, classic retention involves more aspects of traditional assimilation and acculturation. The heterogeneity in retention factors differentially influences immigrants’ sense of belonging as they continue to live and age in Wisconsin, either accepting or rejecting the assimilation process. Our findings underscore the fluidity of transnational identities and how sense of social cohesion and perceived well-being shape older Latino immigrant health behaviors, resulting in disparate health outcomes.

